# Analysis of metabolites of fungal balls in the paranasal sinuses

**DOI:** 10.1186/s12879-022-07710-x

**Published:** 2022-09-13

**Authors:** Xiaoqing Zhang, Na Zhang, Qian Huang, Shunjiu Cui, Lingyan Liu, Bing Zhou

**Affiliations:** 1 Department of Otolaryngology-Head and Neck Surgery, Beijing Tongren Hospital, Capital Medical University, No. 1 Dongjiaominxiang, Dongcheng District, Beijing, 100730 China; 2 Department of Otolaryngology-Head and Neck Surgery, Qinhuangdao First Hospital, Hebei Medical University, Shijiazhuang, Hebei Province China; 3 School of Pharmaceutical Sciences, Capital Medical University, Beijing, 100069 China

**Keywords:** Fungal ball sinusitis, Metabolomics, Glycerophospholipid, Sphingolipid metabolism

## Abstract

Fungal ball sinusitis is characterized by complex fungus infections with non-invasive inflammation. But no research reported fungal ball composition and metabolic-related product types currently. 12 patients with chronic rhinosinusitis who underwent surgery and 9 healthy control were enrolled in this study. Samples from both groups were analyzed for high-throughput metabolites by UPLC-MS. OsiriX software was applied to perform imaging measurements on sinus CT. 2138 and 394 metabolites were screened from cationic and anionic modes. There was a significant difference in the abundance of glycerophospholipid metabolism and sphingolipid metabolism between the two groups, with the experimental group showing an increased trend related to the sphingolipid metabolic pathway, including sphingosine 1-phosphate (S1P) and related products, diacylglycerol, sphingomyelin (SM), suggesting that its metabolites are associated with mucosal and bony inflammation. Imaging measurements showed a median sinus CT value (median (P_25_, P_75_) of 351(261.4, 385.8) HU and a median sinus wall thickness (median (P_25_, P_75_) of 2.31(1.695, 3.718) mm, which correlated with the levels of glycerophospholipid metabolites and sphingolipid metabolites (P < 0.03). Dysfunctional glycerophospholipid and sphingolipid metabolism is present in the lesion of fungal ball sinusitis. Glycerophospholipid and sphingolipid metabolism plays a significant role in the progression of mucosal and osteitis produced by fungal ball sinusitis.

## Introduction

The fungal ball of the paranasal sinus is defined as a non-invasive accumulation of dense fungi in the sinus cavity, most often in the maxillary sinus [1]. Clinical symptoms are nonspecific, including nasal discharge, nasal obstruction, headache, and facial pain [2]. Computed tomography (CT) and magnetic resonance imaging (MRI) have more characteristic performances. CT shows a high-density area, sclerosis of the lateral sinus wall, erosion of the inner sinus wall, and irregular material surface. MRI illustrated a low-intensity area on T2-weighted MR imaging. Imaging examinations indicated high sensitivity and specificity [3–5], suggesting potential mucosa and bone inflammation factors. In the previous studies, the pathogenesis of fungal sinusitis was related to innate mucosal immunity. The fungal colonization pattern molecules continued stimulating the pattern recognition receptors, which activated innate immune cells to synthesize many innate immune molecules. In this way, the apical junction complex in the nasal mucosa of immunodeficient or potentially immunodeficient individuals and atopic individuals exacerbated fungal antigen exposure. As a result, the innate and adaptive immune was triggered and failed [6–8]. Toll-like receptors (TLRs) and C-type lectin-like receptors (CLRs) detect fungal components and activate epithelial cells to release innate inflammatory cytokines including interleukin (IL)-1b, IL-25, IL-33, and thymic stromal lymphopoietin (TSLP). Th2 cells cause eosinophil recruitment, goblet cell hyperplasia, mucus production, and antigen-specific IgE synthesis by secreting IL-4, IL-5, and IL-13 in response to fungal antigens, hence defining type 2 adaptive immune responses [9, 10]. However, there is still a lack of large-scale prospective studies on the pathogenesis of fungal sinusitis, and there is no unified conclusion.

Metabolomics provides the potential to analyze biochemical changes in disease pathology. As the use of metabolomics technology continues to evolve in all drug discovery and development areas, its range of applications continues to expand, and its impact is rapidly expanding [11]. Involving detailed experimental analysis of the metabolic profile, the level of detection can be considered the final response of the biological system, reflecting the overall outcome of the natural event. UPLC/MS (Ultra-performance liquid chromatography coupled to mass spectrometry) is an advanced analytical technology supporting metabolomics and has evolved into a sensitive and highly reproducible platform for the simultaneous determination of hundreds of metabolites [12], widely used in metabolomics research. Nowadays, metabolomics has been commonly used in the study of immune regulation in the intestinal mucosa, but studies in upper airway diseases are relatively limited.

To the best of our knowledge, there are currently no related articles reporting fungal ball composition and metabolic-related product types. Therefore, the objective of this study is to determine the relationship between the composition of the fungal ball and the development of the disease pathogenesis by exploring the metabolic components and to explore the relationship between the composition and clinical features.

## Patients and methods

### Participants

The population comprised 12 adults with fungal balls patients in the maxillary sinus and 9 healthy control. The institutional review board approved the protocol (2019A057) and consent was obtained from all subjects. Demographic patient data and results of serum biochemical examination were recorded. Exclusion criteria included: (1) endoscopic sinus surgery history; (2) severe nasal or maxillofacial trauma history; (3) combined other diseases sinus diseases, including chronic bacterial sinusitis, nasal polyps, tumors, or atopic inflammation; (4) respiratory infection in 3 months; (5) systemic metabolic bone diseases, including diabetes and immunosuppression; (6) applied steroid nasal spray, oral methylprednisolone, antibiotics or immunosuppressants in 2 weeks. Each patient in the research group received ESS from authors Q.H. and S.C, which procedure has been described by Kennedy [13, 14]. With a 0° endoscope, uncinate process resection and anterior ethmoidectomy were conducted, and maxillary sinusotomy was performed at 70° endoscope. 4 mm, 40°/60° microdebriders were used in all cases.

### Sample collection

For the fungal balls patients’ group, samples were immediately obtained during surgery and frozen (− 80°). For the healthy control group, secretion was taken from the middle nasal meatus: a swelling sponge (3.5 × 0.9 × 0.4 cm) was cut into 8 equal portions, and 2 of 8 were placed in healthy control individuals’ middle nasal meatus for 10 min. After taking it out, the two tiny swelling sponges were immersed in 1 ml of 0.9% sodium chloride solution at 4 °C for 2 h and centrifuged at 1500*g* for 15 min at 4 °C. The centrifuged liquid was kept frozen (− 80°) as well. All individuals had twice-daily nasal irrigation for 3 days before collecting samples.

### UPLC-MS experiments

**Fig. 1:**
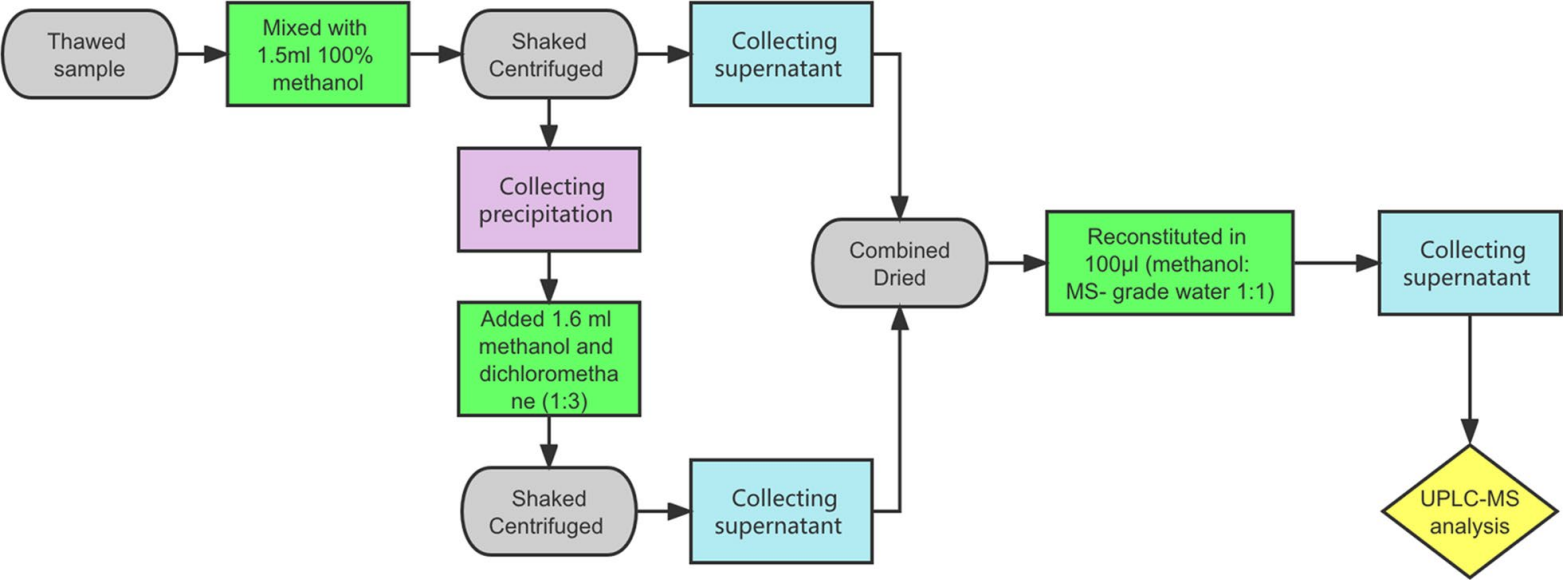
The schematic diagram of the experimental procedures

Each frozen sample was thawed, and a 50 ± 5 mg aliquot was mixed with 1.5 ml 100% methanol (methanol: water = 1:1) in a 2 ml micro-centrifuge tube. The mixture was shaken for 1 h and then centrifuged for 10 min. The precipitation and supernatant were collected for each sample. Then a total of 1.6 ml of methanol and dichloromethane (1:3) was added to the precipitate. Every sample was vibrated and sonicated for 1 h, then centrifuged to obtain the supernatant. The supernatant from both steps was combined and then dried under nitrogen gas flow. Before UPLC-MS analysis, the dried extract was reconstituted in 100 μl (methanol: MS-grade water = 1:1), and centrifuged again to remove any insoluble residuals. Finally, the supernatant was transferred to a sampling bottle. A schematic diagram of the experimental procedures is shown in Fig. 1.

### CT measurement

The anatomical dimensions of all patients were measured using an OsiriX® (Pixmeo, Geneva, Switzerland) viewer in combination with preoperative 3D CT software. Reconstruction parameters and methods such as window width, window level, and head position normalization are consistent with previous studies (Brilliance 64 CT machine, Philips Medical System, Cleveland, OH; scan parameters: 120 kV; 300 mA; matrix size 512 × 512; axial slice thickness 1 mm; window width: × 4000 Hounsfield units (HU); window level: 700 HU) [15, 16].

**Fig. 2:**
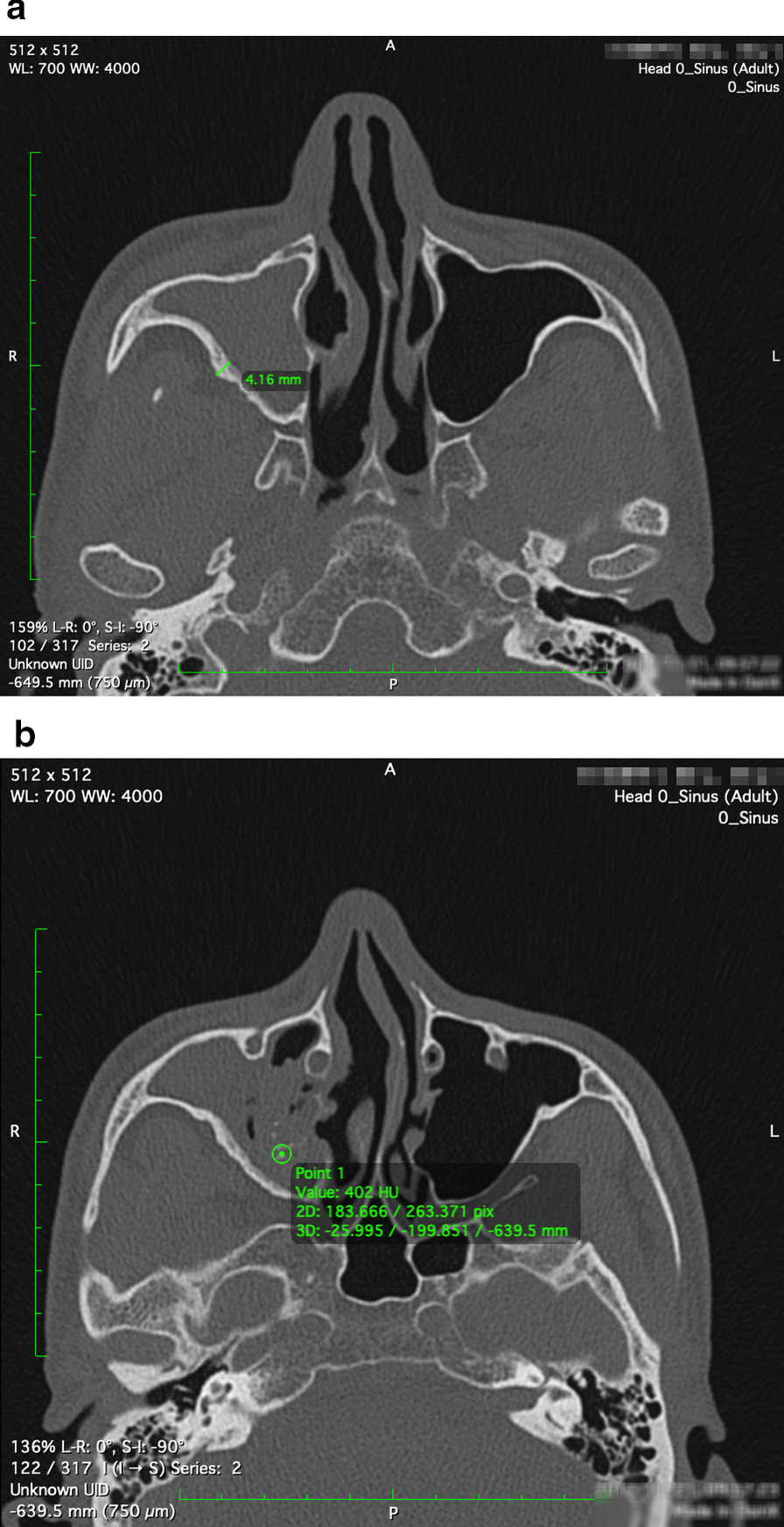
Sinus CT measurements were performed by OsiriX® software. **a** The thickness of the posterior lateral wall of the maxillary sinus; **b** the highest CT value of the high-density area in the maxillary sinus

From the axial view, two dimensions were measured (Fig. 2): (1) thickness of the posterior lateral wall of the maxillary sinus (Fig. 2a); the posterior lateral wall of the maxillary sinus was chosen because it is the most fixed area in the maxillary sinus CT image. (2) The highest CT value of the high-density area in the maxillary sinus (Fig. 2b). The measurement was 3 times and took the average value.

### Data analysis

UPLC-MS data were processed by Progenesis QI 2.0 software and Ezinfo 3.0 (Waters), which performed automatic baseline correction, alignment, and peak peaking. The peaks of missing values were removed by the 80% rule [14]. Selected peak indices with accurate m/z and segmentation information were submitted to online library searches, including Human Metabolome Database (HMDB), Kyoto Encyclopedia of Genes and Genomes (KEGG), ChemSpider, and LipidMAPS. Statistical analysis was performed by SPSS 21 software (SPSS Inc., Chicago, IL), including the Kolmogorov–Smirnov test, Student’s t-test or Mann–Whitney U test, and correlation analysis.

## Result

### Patient characteristics

A total of 12 fungal balls in the maxillary sinus and 9 healthy control who satisfied the inclusion and exclusion criteria were enrolled. There was no significant difference in age and gender between the two groups. Details are shown in Table 1.

**Table 1:** Clinical information of fungal ball patients and healthy control

Characteristics	Fungal ball patients	Healthy controls
Amount	12	9
Age, mean ± SD years	52.5 ± 12.39	50.3 ± 10.28
Gender, male:female	3:9	3:6
Specimen side, right:left	6:6	4:5

### Metabolic differences of follicular fluid between fungal ball sinusitis and controls

A total of 21 samples were analyzed with a random sequence under both positive and negative ion modes. The 80% rule was applied to remove the missing value after peak alignment. Orthogonal signal correction partial least statistical analysis (OPLS) was utilized to summarize the most significant population differences in complex multivariate data. A total of 2138 and 394 compound signals were reserved in ESI positive and ESI negative modes, respectively. The p-values were corrected with Benjamini & Hochberg method.

Compound signals with VIP greater than 1 were selected to further screen metabolites by searching databases including KEGG, HMDB, and ChemSpider. The more rigorous filtering strategy was applied: (a) corrected p < 0.05; (b) MS fragment patterns involved in library search; (c) library (HMDB) search score > 40. Finally, twenty-six metabolites with p < 0.05 were retained and presented in Table 2.

**Table 2:** UPLC-MS detected metabolites that varied in PCOS fungal sinusitis with significant difference (corrected p < 0.05)

Metabolite	m/z	Mass error (ppm)	PCOS vs control^c^	p-value (B-H corrected)	HMDB / ChemSpider ID	Class
Phytosphingosine	318.300^a^	− 0.53	↑	2.1E−02	HMDB0004610	Organonitrogen compounds
LysoPC(18:1(11Z))	544.3354^a^	− 4.14	↑	9.9E−03	HMDB0010385	Glycerophospholipids
PE (22:6(4Z,7Z,10Z,13Z,16Z,19Z)/18:0)	776.5623^a^	4.37	↑	1.1E−04	HMDB0009684	Glycerophospholipids
Tryptophanol	184.0728^a^	− 3.29	↑	1.1E−05	HMDB0244977	Indoles and derivatives
*N*-hexadecanoylsphinganine-1-phosphocholine	703.57161^a^	− 4.60	↑	1.1E−05	HMDB0062665	Sphingolipids
SM(d18:1/18:0)	813.6819^a^	− 3.07	↑	3.8E−05	HMDB0062559	Sphingolipids
Lactosylceramide (d18:1/24:1(15Z))	994.7175^a^	− 1.70	↑	1.5E−04	HMDB0004872	Sphingolipids
PC(*O*-16:0/18:2(9Z,12Z)	786.5988_a_	− 2.43	↑	1.1E−02	HMDB0011151	Glycerophospholipids
PC(*O*-16:0/0:0)	963.7126^a^	− 1.124	↑	1.4E−03	CSID142380	Glycerophospholipids
PC(DiMe(11,3)/MonoMe(11,3))	853.5844^a^	2.06	↓	7.2E−07	HMDB0061395	Glycerophospholipids
Galabiosylceramide (d18:1/16:0)	862.6238^b^	− 1.41	↑	4.3E−04	HMDB0004833	Sphingolipids
dl-Mevalonic acid	319.13698^a^	2.14	↓	1.0E−05	HMDB0006024	Fatty acyls
Diacylglycerol	729.5670^a^	0.83	↑	1.0E−03	HMDB0242173	Glycerolipids
Harman	365.1777^a^	4.45	↓	6.0E−06	HMDB0035196	Alkaloids
PS(14:1(9Z)/14:0)	700.4173^a^	1.91	↓	8.0E−07	HMDB0012341	Glycerophospholipids
PG(22:6(4Z,7Z,10Z,13Z,16Z,19Z)/20:2(11Z,14Z))	869.5322^a^	2.26	↓	7.6E−08	HMDB0116601	Glycerophospholipids
PGP(i-13:0/a-25:0)	909.5572^a^	− 2.33	↓	7.1E−06	HMDB0116553	Glycerophospholipids
CL(8:0/13:0/17:0/18:2(9Z,11Z))	1259.8071^a^	1.70	↓	4.0E−04	HMDB0119867	Glycerophospholipids
*N*-lactoyl-Tryptophan	299.1006^a^	1.34	↑	2.9E−04	HMDB0062178	Carboxylic acids and derivatives
Ganglioside GM3 (d18:0/12:0)	1099.6683^a^	− 4.73	↓	2.1E−06	HMDB0011913	Glycosphingolipids
l-Phenylalanine	166.0863^a^	0.25	↑	6.5E−03	HMDB0000159	Carboxylic acids and derivatives
PGP(18:3(9Z,12Z,15Z)/16:0)	823.4563^b^	3.80	↓	4.7E−04	HMDB0013577	Glycerophospholipids
LysoPC(0:0/18:2(9Z,12Z))	542.3214^a^	− 2.86	↑	1.2E−02	HMDB0061700	Glycerophospholipids
Tenuazonic acid	395.2179^a^	0.58	↑	5.9E−13	HMDB0036074	Pyrrolines (Fungal toxins)
Farnesyl pyrophosphate	383.1399^a^	4.17	↓	1.3E−03	HMDB0000961	Prenol lipids
1-Oleoylglycerophosphoinositol	599.3181^a^	− 1.66	↓	7.7E−15	HMDB0061693	Glycerophospholipids

^a^Metabolites were detected in positive ion mode; ^b^metabolites were detected in negative ion mode; ^c^Arrows indicate increase (↑) or decrease (↓) in the fungal balls compared with secretion of healthy people

From ESI positive mode, levels of LysoPC (Lysophosphatidylcholine, Substituents: 1-acyl-*sn*-glycero-3-phosphocholine), PE (phosphatidylethanolamine), PC (phosphocholine), 2-linoleoyl-*sn*-glycero-3-phosphocholine (Substituents: 2-acyl-*sn*-glycero-3-phosphocholine) in patients with fungal balls sinusitis were increased, while levels of PS (phosphatidylserine), PG (phosphatidylglycerol), PGP (phosphatidylglycerophosphate), CL (cardiolipin) showed decreased. All sphingolipids indicated significantly higher than healthy controls, including *N*-hexadecanoylsphinganine-1-phosphocholine (Sphingoid-1-phosphate or derivatives), Sphingomyelin, Lactosylceramide, Galabiosylceramide, Phytosphingosine.

### Disturbance in metabolism of fungal balls

**Fig. 3:**
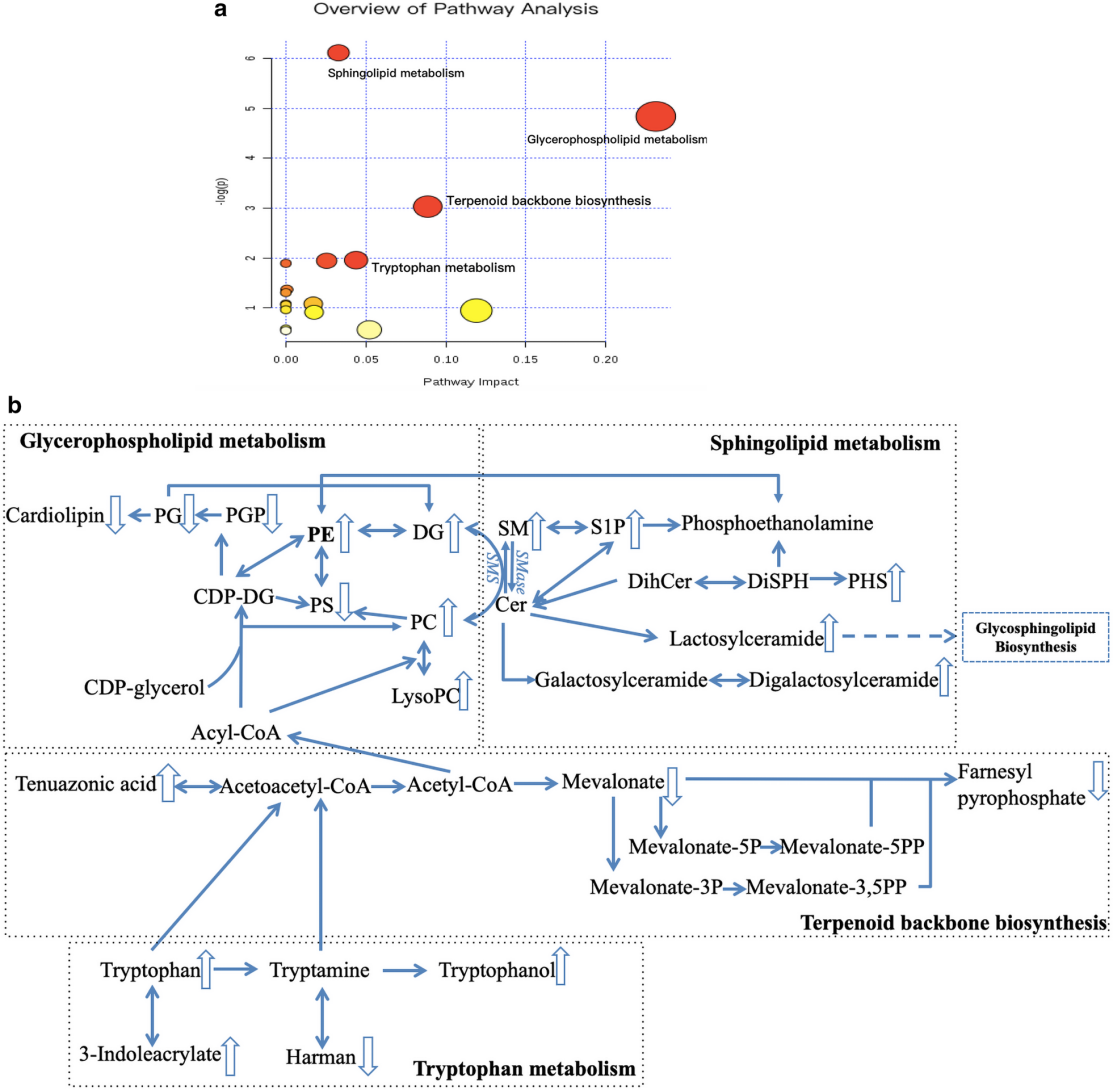
**a** Overview pathway enrichment analysis for metabolites suggested by MetPA. **b** Metabolic pathway diagram showing altered metabolites in PCOS with the fungal ball. An upward (downward) arrow indicated a significantly higher (lower) level in the metabolite list (p < 0.05). PE: phosphatidylethanolamines; DG: diacylglycerol; CDP-DG: CDP-diacylglycerol; PG: phosphatidylglycerol; PGP: phosphatidylglycerolphosphate; PS: phosphatidylserine; PC: phosphatidylcholine; LysoPC: lysophosphatidylcholine; S1P: sphingosine-1-phosphate; SM: sphingomyelin; Cer: ceramide; SPH: sphingosine; DihCer: dihydroceramide; DiSPH: dihydrosphingosine

The online resource MetPA was utilized to locate metabolites in pathway maps that were associated with the fungal balls group. Figure 3a showed all matched pathways according to the p values of the pathway enrichment analysis, and pathway impact values of the pathway topology analysis generated by MetPA. It indicated that the impacts of glycerophospholipid metabolism and sphingolipid metabolism were most remarkable. Figure 3b presented the complete map involving 4 pathways with significant differences in the fungal ball group. Metabolites with arrows were explored by UPLC-MS and met further screen conditions. Most metabolites from glycerophospholipid metabolism showed a downward trend, whereas those from sphingolipid metabolism presented upward. Tryptophan metabolism and terpenoid backbone biosynthesis were associated with the glycerophospholipid metabolism and sphingolipid metabolism pathway via Acyl-CoA.

### Correlations between altered metabolites and clinical characteristics in the fugal ball group

The CT measurements were as follows: (1) the median value of the thickness of the posterior lateral wall of the maxillary sinus in fungal balls was 2.31 (P_25_, P_75_, 1.695, 3.718) millimeters; (2) the median highest CT value of the high-density area in the maxillary sinus of fungal balls was 351(P_25_, P_75_, 261.4, 385.8) Hounsfield Unit (HU). *Spearman* correlation coefficient was applied to analyze the median value of the thickness of the posterior lateral wall of the maxillary sinus and the median highest CT value of the high-density area in the maxillary sinus with metabolites, respectively. Levels of phytosphingosine, PE (22:6(4Z,7Z,10Z,13Z,16Z,19Z)/18:0) presented a positive correlation with a median value of the thickness of the posterior lateral wall of the maxillary sinus. At the same time, PC(O-16:0/0:0) was correlated positively with the CT value of the high-density area in the maxillary sinus. For serum biochemical results, dl-Phenylalanine was associated with the concentration of calcium ions (Table3).

**Table 3:** *Spearman* correlation coefficients between UPLC-MS detected metabolites of fungal balls and clinical results in patients (p < .03)

Clinical information/Metabolites	Patients’ result (median, P_25_, P_75_)	Correlation coefficient
Thickness of the posterior lateral wall of maxillary sinus	2.31 (1.695, 3.718) mm	
PC(*O*-16:0/0:0)		0.721*
Highest CT value of high-density area in maxillary sinus	351 (261.4, 385.8)HU	
Phytosphingosine		0.699
PE(22:6(4Z,7Z,10Z,13Z,16Z,19Z)/18:0)		0.772*

^*^The *Spearman* correlation p-value was illustrated if it is less than 0.03

## Discussion

Fungal sinusitis is a typical unilateral lesion in the clinic. It has been reported that the levels of IgA, plasma cells, and lymphocytes in the secretion of fungal sinusitis are elevated [17]. In contrast, other inflammatory cells are not infiltrated, suggesting that mucosal immunity induced by fungal sinus balls plays a vital role in the progression of the disease [17]. In addition to the unilateral soft tissue density, shadow in the imaging examination can also be expressed as sinus wall bone changes. It is undeniable that the content of the sinus in fungal sinusitis (fungal mass, sinus secretion) is related to clinical manifestations. Still, due to technical limitations, few pieces of literature describe the composition of sinus contents and their clinical relationship. In the past few decades, with the development of science and technology and the concept of "omics", metabolomics-related techniques have made possible component analysis for complex samples. In this study, we first explored the metabolic differences of fungal sinusitis using metabolomics and initially linked them with clinical manifestations.

Twenty-six metabolites were found related to the disease. In this study, the vast majority of the molecules with putative identification were lipids. Glycerophospholipids were the most detected metabolites associated with the fungal ball. The cell membrane of fungi is enriched with diverse lipids belonging to the class glycerophospholipids, sphingolipids, and sterols. Glycerophospholipids serve as a stable structural component of biological membranes with *sn*-3 configuration of the glycerol backbone, playing a pivotal role in regulating transport, signal transduction, and protein function regulation [18]. PC and PE are essential and represent the major components of both cellular and subcellular membranes. The decrease of PC is related to the degradation of microbial cells in a fungal ball. PE can also be formed via calcium-dependent head group exchange with pre-existing phospholipids [19]. This study showed that PE level was positively correlated with osteogenesis but negatively correlated with serum calcium ion level. It is speculated that PE participates in key metabolites of sinus wall bone changes. PE is required for energy-dependent severe substrate accumulation. Sinus wall bone hyperplasia in sinus CT is one of the typical features of fungal rhinosinusitis, and we speculate that it may be related to PE metabolism. However, there is still a lack of in-depth research on its mechanism.

**Fig. 4:**
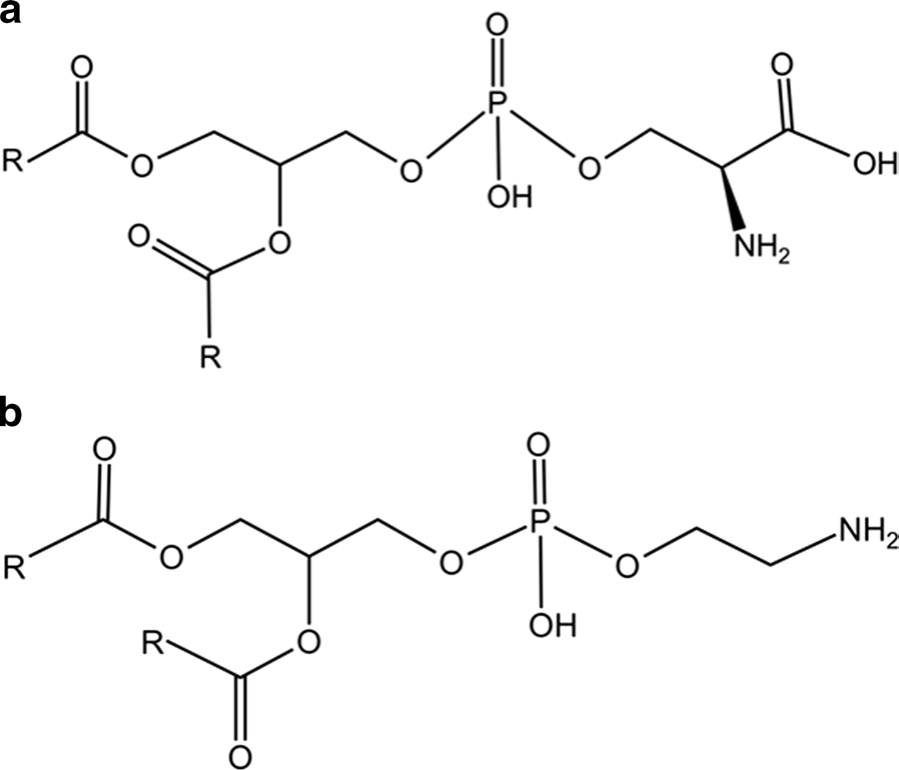
Molecular structure diagram of **a** phosphatidylserine (PS) and **b** phosphatidylethanolamine (PE). R represented a hydrocarbon or alkyl group in the molecular structure diagram

Both phosphatidylserine (PS) and phosphatidylethanolamine (PE) were connected to the diglycerides of the sphingolipid metabolic pathway through metabolic conversion (Fig. 4). The conversion between the three regulates cell differentiation, proliferation, and apoptosis in other diseases, including Alzheimer's disease, atherosclerosis, chronic inflammation, etc. [20–22]. The role of the base bridge is to connect the protein on the plasma membrane through sugar. Enrichment of lipids in different cell corners could be attributed to acyl chain remodeling, presented in the pathways in this study [23]. Ceramide, sphingosine, and sphingosine 1-phosphate (S1P) are the primary bioactive mediators of sphingolipid metabolism. S1P might be involved in the immune and inflammatory responses of potent cytokines. These ceramide-rich platforms involve various signaling cascades in immune cells, including B cell activation, bacterial pathogen infection, and release of cytokines during infection; they are also essential in inducing apoptosis [24]. Studies have shown that S1P influences bone remodeling [25]. In this study, the content of S1P increased, suggesting that the expression of S1P is related to the bone changes characteristic of fungal sinusitis. In addition, AhR (aryl hydrocarbon receptor) and IDO1(2,3-dioxygenase 1) have been reported to play a vital role in linking the catabolism of microbial tryptophan and host endogenous tryptophan metabolites to regulatory T cell function in the mucosal region [26]. A positive feedback loop between IDO1 and AhR is necessary to drive the co-evolution of symbiotic fungi with the mammalian immune system and microbiota, host survival under stable inflammatory conditions, and fungal symbiosis to prevent immune dysregulation [27], which is consistent with the non-invasive characteristics of the fungal ball. In this study, tryptophan metabolism can elicit both hyper- and anti-inflammatory effects, regulated by Tenuazonic acid (fungal toxins), but the mechanism requires further research. One of the features of metabolomics research is the capacity to qualitatively and quantitatively investigate the link between metabolites and physiopathological changes in the body. In clinical practice, fungal ball sinusitis has the potential to develop into an invasive condition, which frequently results in a poor prognosis. Individuals with contraindications to general anesthesia who are prone to develop invasive fungal sinusitis may find new therapeutic options according to the metabolite analysis results of this study. By interfering with the creation of sphingolipid metabolism and tryptophan metabolism, this study's analysis of metabolites may reveal fresh techniques for treating the illness. To confirm the findings, the design of future in vitro and in vivo experiments needs to be refined.

We tried to correlate metabolites and imaging. As a principal constituent of the lipid fraction present in the calcification front during normal bone formation, the thickness of the posterior lateral wall of the maxillary sinus was positively correlated with the PC (O-16:0/0:0) level, indicating PC had a boosting effect on osteogenesis. Li et al. found that PC metabolism in human osteoblasts and its metabolites contribute to the growth and mineralization of human osteosarcoma cells through metabolomic studies [28]. PC also could affect the osteogenic transdifferentiation of vascular smooth muscle cells into calcified vascular cells [29]. On the other hand, the highest CT value of the high-density area in the maxillary sinus was positively correlated with phosphatidylethanolamine, a key metabolite in the interconversion of glycerophospholipid metabolism and sphingolipid metabolism to augment inflammatory signaling.

The following limitations exist in this study. Firstly, the metabolomic differences between different groups of fungi were not obtained by diversity analysis of fungi; secondly, the relatively small sample size may have biased the selection. Further studies should include an analysis of microbial community composition and the distribution of large samples.

## Conclusion

In this study, we applied UPLC/MS to analyze the metabolic components of fungal balls to speculate metabolic pathways, trying to explain the pathogenesis of the disease from a metabolomics perspective. Dysfunctional glycerophospholipid and sphingolipid metabolism was present in patients with fungal ball sinusitis. Glycerophospholipid and sphingolipid metabolism contributed negligibly to the progression of mucosal and osteitis that arises in fungal ball sinusitis. Future work could explore the relationship between biodiversity and the fungal ball, and new treatment ideas for the disease could be suggested based on these findings.

## Data Availability

The dataset generated and/or analyzed in the current study, is not public due to its involvement in another unpublished more in-depth study, but is available from the corresponding author on reasonable request.

## Abbreviations

Cer: Ceramide
CL: Cardiolipin
CT: Computed tomography
DG: Diacylglycerol
DihCer: Dihydroceramide
DiSPH: Dihydrosphingosine
HMDB: Human Metabolome Database
HU: Hounsfield Unit
KEGG: Kyoto Encyclopedia of Genes and Genomes
LysoPC: Lysophosphatidylcholine
MRI: Magnetic resonance imaging
PC: Phosphocholine
PE: Phosphatidylethanolamine
PG: Phosphatidylglycerol
PGP: Phosphatidylglycerophosphate
PS: Phosphatidylserine
S1P: Sphingosine-1-phosphate
SM: Sphingomyelin
SPH: Sphingosine
UPLC/MS: Ultra-performance liquid chromatography coupled to mass spectrometry
